# I2N: image to nutrients, a sensor guided semi-automated tool for annotation of images for nutrition analysis of eating episodes

**DOI:** 10.3389/fnut.2023.1191962

**Published:** 2023-07-27

**Authors:** Tonmoy Ghosh, Megan A. McCrory, Tyson Marden, Janine Higgins, Alex Kojo Anderson, Christabel Ampong Domfe, Wenyan Jia, Benny Lo, Gary Frost, Matilda Steiner-Asiedu, Tom Baranowski, Mingui Sun, Edward Sazonov

**Affiliations:** ^1^Department of Electrical and Computer Engineering, University of Alabama, Tuscaloosa, AL, United States; ^2^Department of Health Sciences, Boston University, Boston, MA, United States; ^3^Colorado Clinical and Translational Sciences Institute, University of Colorado, Denver, CO, United States; ^4^Department of Medicine, University of Colorado Anschutz Medical Campus, Aurora, CO, United States; ^5^Department of Nutritional Sciences, University of Georgia, Athens, GA, United States; ^6^Department of Electrical and Computer Engineering, University of Pittsburgh, Pittsburgh, PA, United States; ^7^Department of Surgery and Cancer, Imperial College, London, United Kingdom; ^8^Department of Metabolism, Digestion and Reproduction, Imperial College, London, United Kingdom; ^9^Department of Nutrition and Food Science, University of Ghana, Accra, Ghana; ^10^Children’s Nutrition Research Center, Department of Pediatrics, Baylor College of Medicine, Houston, TX, United States; ^11^Department of Neurological Surgery, University of Pittsburgh, Pittsburgh, PA, United States

**Keywords:** food intake, image annotation, image to nutrients, software, wearable sensor

## Abstract

**Introduction:**

Dietary assessment is important for understanding nutritional status. Traditional methods of monitoring food intake through self-report such as diet diaries, 24-hour dietary recall, and food frequency questionnaires may be subject to errors and can be time-consuming for the user.

**Methods:**

This paper presents a semi-automatic dietary assessment tool we developed - a desktop application called Image to Nutrients (I2N) - to process sensor-detected eating events and images captured during these eating events by a wearable sensor. I2N has the capacity to offer multiple food and nutrient databases (e.g., USDA-SR, FNDDS, USDA Global Branded Food Products Database) for annotating eating episodes and food items. I2N estimates energy intake, nutritional content, and the amount consumed. The components of I2N are three-fold: 1) sensor-guided image review, 2) annotation of food images for nutritional analysis, and 3) access to multiple food databases. Two studies were used to evaluate the feasibility and usefulness of I2N: 1) a US-based study with 30 participants and a total of 60 days of data and 2) a Ghana-based study with 41 participants and a total of 41 days of data).

**Results:**

In both studies, a total of 314 eating episodes were annotated using at least three food databases. Using I2N’s sensor-guided image review, the number of images that needed to be reviewed was reduced by 93% and 85% for the two studies, respectively, compared to reviewing all the images.

**Discussion:**

I2N is a unique tool that allows for simultaneous viewing of food images, sensor-guided image review, and access to multiple databases in one tool, making nutritional analysis of food images efficient. The tool is flexible, allowing for nutritional analysis of images if sensor signals aren’t available.

## 1. Introduction

According to the World Health Organization (WHO), > 2.8 million people worldwide die annually due to being overweight or obese (1). Excess body weight accumulates as the result of a long-term energy imbalance such that calories consumed are less than expended over time (2). Thus, an objective assessment of caloric intake is critical as a first step in addressing obesity. Additionally, assessment of dietary intake is important for understanding the nutritional status and overall health.

Traditional dietary assessment methods include food diaries, 24 h dietary recalls, and food frequency questionaries (FFQs) (3). Most of these methods rely on participants’ recollection and declaration (self-report) and, thus, may suffer from underreporting, misreporting, and non-reporting of caloric intake (4). Owing to its high accuracy, the doubly labeled water (DLW) method is the gold standard for assessing total energy expenditure in community-dwelling people (5). DLW can be used to measure energy intake by using the intake balance method (6). The intake balance method determines true energy intake by taking changes in body weight or body energy stores into account. However, this method requires biological samples (e.g., urine) to be collected at a specific interval, which must be analyzed by an expert technician using a specialized measurement instrument, making this method expensive and unfeasible for day-to-day application, and it provides no information on specific foods consumed.

In recent years, dietary assessment methods that include image capture and/or sensors have been developed (7–12). While these methods are still relatively new and are continually undergoing improvement, they offer an advantage over traditional dietary assessment methods which are subjective and rely on self-report. Image-capture and sensor technologies are more objective, which can help overcome self-report bias. However, to our knowledge, there are no tools designed exclusively for annotation and nutritional analysis of images that can also incorporate sensor data and easy access to existing and widely used food and nutrient databases such as FNDDS (13), USDA-SR (14), and others.

In this study, we describe and evaluate a sensor-guided semi-automated image reviewing tool, Image to Nutrients, or I2N, that can provide energy and nutrient information. I2N eliminates the need for the user to recall or report food intake episodes. I2N is a desktop software based on Java programming that displays sensor-detected eating episodes and images and accesses multiple food and nutrient databases for calculating energy and nutrient composition of foods and eating episodes. An individual examines the images uploaded into the software to identify food items and quantify their portion sizes. Energy intake and nutrient values, including macronutrients and micronutrients, are provided by I2N. I2N also creates reports on the number of eating episodes, the number of food items consumed, the weight of food consumed, and the energy and nutrients consumed by food, eating occasion, and day. I2N provides a convenient way to conduct nutritional analysis of food images, with or without accompanying sensor data.

## 2. Methods

This paper presents a semi-automatic image annotation tool, I2N, for the assessment of dietary intake. We used Java (a computer programming language), to create this desktop application. The application was developed to be used with images and sensor data collected by the Automatic Ingestion Monitor Version 2 (AIM-2) (10–12); however, I2N could be used with food images collected from other wearable or non-wearable devices after the images undergo processing to a format that can be uploaded into the software. The description of the wearable sensor system, data collection, raw image processing, automatic food intake detection, and I2N annotation tool is detailed below.

### 2.1. Wearable sensor system

The AIM-2 (15–17) was the primary source of data used in the studies described below. The AIM-2 is equipped with a 5-megapixel camera, a USB port, a three-axis accelerometer, and an optical eating detection sensor. A fully charged battery can record data for more than 24 h on a single charge of battery, and the battery can be recharged to allow for collection of additional data over more days. A plastic enclosure protects the sensors and electronics. The AIM-2 attaches to the right side of the frame of a pair of eyeglasses (if an individual does not wear eyeglasses, a pair of non-corrective eyeglasses is provided). The accelerometer and the optical sensor signals are captured at a sample rate of 128 Hz. The camera captures egocentric images (i.e., images taken by a wearable camera from the user’s viewpoint) periodically at a rate of one image per 10 or 15 s (this parameter is programmable). The attachment of the AIM to eyeglasses means the camera is always aligned with eye gaze as long as the eyeglasses are worn as usual, which allows the images to capture the foods from the same perspective as our visual perception when we are eating. All sensor signals and images are stored on an internal SD card. The image and signal are collected passively, which means that the user’s active participation is not required. Here, the accelerometer records head movements and leaning forward movement, the optical sensor records temporalis muscle movement (related to eating activity), and the camera captures egocentric view images (provides consumed food images).

### 2.2. Automatic food intake detection

The AIM-2 detects eating episodes using models from our previous work (12, 15). Sensor data were used to extract features (e.g., slope sign change, zero crossing rate), and a support machine vector (SVM) classifier was trained to detect food intake epochs (e.g., 8 s) (15), it is to be mentioned that sensor signal was collected continuously with a sampling frequency of 128 Hz. Detected epochs were used to compute the boundaries of eating episodes (i.e., beginning and end). Each image is labeled as 0 or 1, where 1 is for food intake and 0 for non-food intake events using the method developed in Doulah et al. (15). The output of the automatic food intake detection algorithm is the sensor-detected eating episode (SDEEp). These detected eating episodes provide guidance in reviewing images associated with eating events.

### 2.3. I2N annotation tool

#### 2.3.1. Inputs

**Figure 1:**
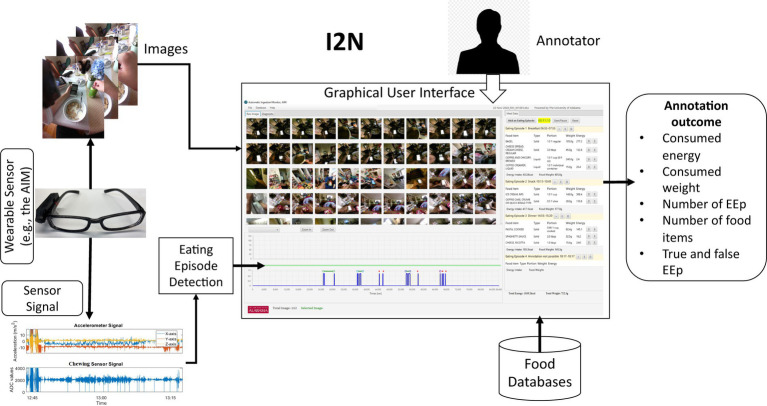
Image to Nutrients (I2N) software architecture.

I2N used the images captured by the AIM-2 and labels from the eating episode detection. Images were processed using the method described in Raju and Sazonov (18) before being loaded into the I2N. The location of the image folder on the local drive and the labels for food intake detection were saved in a data file called the project file, which was directly loaded into the I2N. The use of sensor eating detection allows the annotator (person reviewing the images) to review only those images that belong to an eating event and ignore images captured in other activities of daily living. The sensor-detected eating episodes are used as input to I2N. In addition, the images captured on the same day are loaded into the I2N. Both the overall architecture and the inputs involved in I2N are illustrated in Figure 1.

#### 2.3.2. Nutrient databases

Standard food and nutrient databases can be used to annotate food items in I2N and conduct nutritional analysis. These databases include the name/description of the food, its corresponding portion size, energy, and nutrient information. The databases are each freely available and can be downloaded in .csv format from their respective websites. Any combination of the available databases may be used in the analysis. We did not perform standardization across the databases, as these mostly contain composite food items and the nutrient values reflect local cooking recipes.

1. USDA National Nutrient Database for Standard Reference (14): The National Nutrient Database for Standard Reference (SR) of the United States Department of Agriculture (USDA) is the primary source of food composition data in the country, and this database serves as the foundation for most food composition databases in the public and private sectors. This database was published in 2018 and is called SR-Legacy (standard reference legacy), which is a successor of SR28 (2015). SR-Legacy contains data on 7,793 food items and up to 150 food components. All the nutrient contents are reported on 100 grams weight of that food items. For simplicity, in this paper, this database is called USDA-SR. The USDA-SR encompasses a wide range of food items, including single ingredients, whole foods, select brand name items, and a limited number of prepared dishes.
2. Food and Nutrient Database for Dietary Studies (FNDDS) by USDA (13). FNDDS contains foods and beverages reported in What We Eat in America (WWEIA) and the National Health and Nutrition Examination Survey (NHANES) in gram amounts and calculates nutrient values for them. We used the 2017–2018 version of FNDDS. The FNDDS contains 7,083 food and beverage items (6,286 foods/792 beverages). Nutrient values per 100 grams of edible portion for energy and 64 nutrients/food components for each FNDDS food/beverage item are found in this database. The FNDDS offers a more comprehensive collection of food items, consisting of whole foods, select brand name products, and a greater variety of prepared dishes not found in the USDA-SR database.
3. USDA Global Branded Food Products Database (BFPD) (19): The USDA Branded Food Products Database is the outcome of a Public-Private Partnership aimed at improving public health and open data sharing by supplementing the USDA Food Composition Databases with the nutrient composition of branded goods and private label data given by the food industry. There were a total of 239,089 food items or private labels listed in this database. This database was used for annotating restaurant and brand food items.
4. Kenyan food composition table (20): Kenyan food composition table was published in 2019 by the Government of Kenya and the Food and Agriculture Organization of the United Nations (FAO). This table contains 142 composite foods and their corresponding nutrient information, including energy in kcal, protein, fat, carbohydrate, vitamins, and minerals.
5. Ugandan food composition table (21): Uganda’s food composition table represents a compilation of existing and imputed food composition data for foods commonly consumed in central and eastern Uganda. This table was published in 2012 and contains 511 composite food items.
6. Food composition table for Western Africa (22): The FAO/INFOODS Western African Food Composition Table (WAFCT 2019) is an update of the 2012 West African Food Composition Table, which lacked some key components, foods, and recipes. WAFCT 2019 has over three times as many food entries and twice as many components as the 2012 version, with improved data quality overall. This table has 1,028 composite food items.

#### 2.3.3. Software architecture

**Figure 2:**
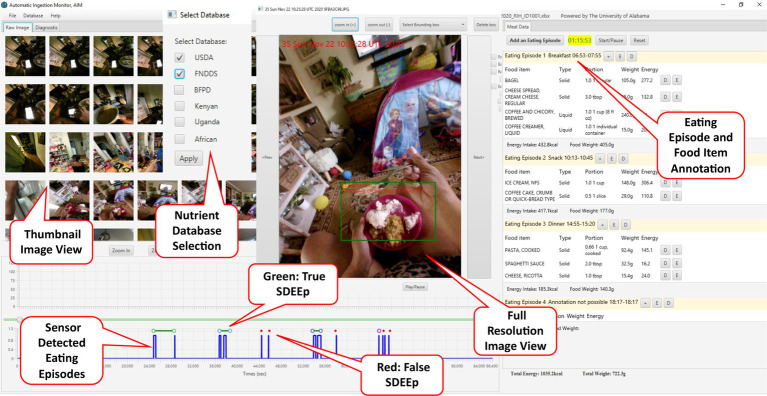
Overview of the I2N graphical user interface.

A graphical user interface (GUI) allows the annotator to interact with images and sensor detected eating episodes. The GUI is intended to facilitate the annotation of eating events for nutritional analysis, including allowing for the naming of the event (e.g., breakfast, lunch, dinner, snack); indication of the start and end times of the eating episodes by linking with the image time stamps; marking the food form (solid, liquid, or semi-solid); identifying food and beverage items, selecting database item from the nutrient databases; and indicating the initial portion, and, if any, the leftover portion. Figure 2 represents an overview of the GUI. The sensor detected eating episodes are plotted on the bottom left side of the GUI. The image panel (top left side of the GUI) shows the thumbnails of images collected during the day. By scrolling the image panel, the annotator can see all the images. The SDEEps guide the annotator in reviewing the images collected during food intake. The annotator can also view a full-resolution image by double-clicking on the thumbnail image. A new window will open, displaying a larger image. The SDEEps indicate eating episodes, but some of these may be false eating episodes, meaning the automatic food intake detection algorithm incorrectly detected eating episodes in which no food image was captured. The number of false eating episodes is expected to be relatively low, since our previous work shows a high rate of accuracy of eating episode detection of 87% based on the F1 score (8, 15) with only 17.3% false negative rate. If the annotator finds food in the larger image (full resolution), then the annotator labels the SDEEp as a true eating episode. On the other hand, if the annotator does not find food in the larger image, then the annotator labels the SDEEp as a false eating episode. After detecting a true eating episode, the annotator adds an eating episode (called an annotated eating episode, AEEp) to the meal panel (right most panel of the GUI). Each AEEp may be given a label (e.g., breakfast, lunch, dinner, or snack), start-time, and end-time. Under AEEp, the annotator adds database items appearing in the images. I2N allows the annotator to search the desired nutrient databases by the food item name. The annotator selects the desired food item and its corresponding portion size (e.g., cup, tablespoon, gram, etc.). Nutrient databases provide portion-size options for a selected food item. Next, the annotator estimates the initial and leftover portion size of the selected food item. After getting all these inputs from the annotator, I2N calculates the amount consumed and the energy and nutrient contents. Moreover, in order to annotate food items, the annotator can choose a single or multiple nutrient database (Figure 2) to find the best match for each food item. After completing the annotation, the annotator can save the annotation output. It is recommended that the annotator establish a standard protocol for annotating images, as described recently (23).

**Figure 3:**
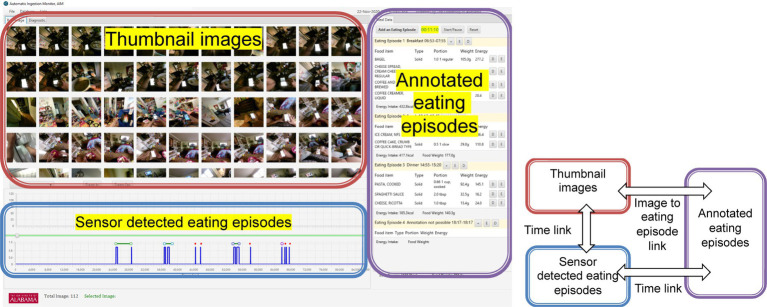
Data flow among panels.

I2N provides the annotator with numerous features that make the task simple, convenient, and time efficient. One of the most important features of I2N is the time link; there is a time link between the thumbnail images and SDEEp, as well as between the SDEEp and start- and end-time of AEEp. This link is demonstrated in Figure 3. When the annotator selects an image, I2N focuses on the SDEEp that corresponds to the image capture time and vice versa, making it easy to see the images associated with a given eating episode along with the nutritional analysis of that eating episode. Similarly, the start- and end-time of an AEEp are linked to the SDEEp. The image to eating episode link is another crucial link. After reviewing an image, an eating episode (annotated) is entered, and the annotator can associate that image with the entered eating episode (annotated). This link allows annotators to track which AEEps originate from which images.

#### 2.3.4. Outputs of I2N

The outputs of I2N are given in Table 1. Energy and nutrients are key components of the output. Energy consumption is tracked throughout the day by AEEp. The output shows the food consumed, initial, and leftover amount in grams. The number of AEEp, and the start and end times of each AEEp are also shown. The consumed amount of a food item is calculated by subtracting the leftover amount from the initial amount. For example, if the initial amount is 1 cup and the leftover amount is 1/3 cup, then the consumed amount is 1−1/3=2/3 cup. Using the portion weight table of the selected nutrient database, I2N converts the consumed amount to the consumed weight in grams. Similarly, utilizing the consumed weight and nutrient database, I2N calculates energy intake in kcal. Thus, I2N calculates number of food items, consumed energy, and consumed weight in each AEEp, followed by daily energy intake and consumed weight. I2N also reports the amount of consumed nutrients such as protein, lipid, carbohydrates, vitamins, mineral etc. calculated from food and nutrient databases used in the analysis.

**Table 1:** Outputs of I2N.

Term	Description
True SDEEp	Annotator marked a SDEEp as a true if captured images contain food items. I2N provides the total number of true SDEEp and its corresponding time. This validates the SDEEp.
False SDEEp	Annotator marked a SDEEp as false if the captured images of a SDEEp do not contain any food items. This helps to remove false SDEEp from the annotated record.
AEEp summary	AEEp description (breakfast, lunch, snack, dinner)
	Start, and end time of AEEp
	Number of foods/ingredients annotated in AEEp
	Consumed energy in Kcal
	Consumed weight in gram
Food item information	AEEp that contains this food item
	Source nutrient database
	Food ID (comes from nutrient database)
	Type (solid, liquid, semi-solid)
	Group name (food group comes from nutrient database)
	Food item name
	Initial, leftover, and consumed amount and portion
	Energy in kcal
	Nutrient information (from nutrient database)
Daily summary	Total number of AEEp
	Total number of foods/ingredients annotated
	Total consumed amount
	Total consumed energy
Annotation time	Total annotation duration (hh:mm:ss) – amount of time it took to complete the annotation of the full record.

SDEEp, sensor-detected eating episode; AEEp, annotated eating episode; ID, food identification number from database.

### 2.4. Evaluation of I2N

Data from two previous studies, one conducted in the US and another in Ghana, were used to evaluate I2N software for three aspects of performance and feasibility: 1) sensor-guided image review, 2) annotation of food images for nutritional analysis, and 3) access to multiple food databases.

#### 2.4.1. Study design

##### 2.4.1.1. US study

Data collection was carried out at the University of Alabama’s Department of Electrical and Computer Engineering. Thirty healthy participants (female: 10, male: 20) between the ages of 18 and 45 years with no medical conditions that interfered with their ability to eat were recruited. The study was approved by the University of Alabama’s Institutional Review Board, and all participants provided written, informed consent prior to participation. The AIM was programmed to collect an image every 15 s. Each participant wore the AIM-2 for 2 days. On day 1, participants were free to do whatever they liked but had to choose their food from an on-campus food court and eat their meals (breakfast, lunch, and dinner) in a laboratory environment. Day 2 was a free-living day without any restrictions. In total, we collected 60 days of data. These data were used to annotate the eating episodes of each participant using I2N by an experienced annotator.

##### 2.4.1.2. Ghana study

Data from 20 households (10 urban and10 rural) were collected in Mampong-Akuapem, a semi-rural community, and Kweiman, a peri-urban community, in the Eastern and Greater Accra Regions of Ghana. The AIM was programmed to collect an image every 15 s. A total of 41 participants (12 adolescents and 29 adults) wore the AIM-2 for a day while living their usual lives in free-living conditions. There was no restriction on the activity, the number of meals/snacks, or food choices. The human subject data collection in Ghana was approved by the Human Subject Institutional Review Boards of the University of Georgia and the University of Ghana. After collecting and processing the data, the data were annotated by an experienced annotator using I2N.

#### 2.4.2. Evaluation procedure

The collected data from those studies are annotated by the nutritionist. The annotators followed standard food and database selection steps as described in Pan et al. (23). For the US Study, three annotators underwent ten 60-min training sessions during which they analyzed 56 days of eating episodes from the first eight participants. For the Ghana Study, there was one annotator who underwent multiple training sessions using data from a previous study in Ghana. Throughout the training sessions, the nutritionists identified certain best practices that were needed to improve the analysis of passive capture images and reduce variability among nutritionists. These practices included: (a) standardizing food selection *a priori* for commonly consumed, high energy density foods and (b) re-evaluating the nutrient database hierarchy when analyzing images.

The usability of I2N was assessed using a sensor-guided image review. In both studies, the total number of captured images was compared to sensor-detected images (images captured during SDEEp by the automatic algorithm described in section 2.3). The number of images reviewed by the I2N was equal to the number of images detected by the sensor. Furthermore, the percentages of images reviewed by annotators were calculated. This assessment indicated how many images the annotator could skip without reviewing. Additionally, we assessed the number of blurred images that may impact food item recognition using the methodology described in Raju and Sazonov (18).

In both studies, all of the database listed above were available for use by the annotators with the exception of the Ugandan food composition table (21). The nutrition analysis output was saved in a tabular format within a Microsoft Excel file. The excel file contains all of the output mentioned in Section 2.3.3. To determine the need for multiple food databases, all annotated food items were counted to determine the source database. The frequency of database use is expressed as a percentage.

## 3. Results

The number of images reviewed in both the US and Ghana studies is shown in Table 2. The sensor-guided image review reduced the number of images that needed to be reviewed in the US study by 93%, and in the Ghana study 85%, in comparison with the total number of images collected during each study. Blur analysis indicated that an average of 13.77% ± 8.93% of the images were blurred, suggesting that the majority of the images could be used for food identification.

**Table 2:** Number of participants, days of data collection, and images in the US and Ghana studies.

Study	No. participants	No. of days	Total no. of images	No. of sensor detected images	Images reviewed (% of total)
US study	30	60	180,520	12,267	7%
Ghana study	41	41	138,076	20,005	15%

An example annotation output from the US and Ghana study is shown in Supplementary Figure S1. Abbreviated results were shown in a GUI and a more extensive and detailed output were exported as a Microsoft excel file. Each food item is associated with an eating episode number, eating episode description, database information, food type (i.e., food form), amount, portion-size, leftover amount, leftover portion-size, consumed weight in grams, and energy and nutrient intake.

**Figure 4:**
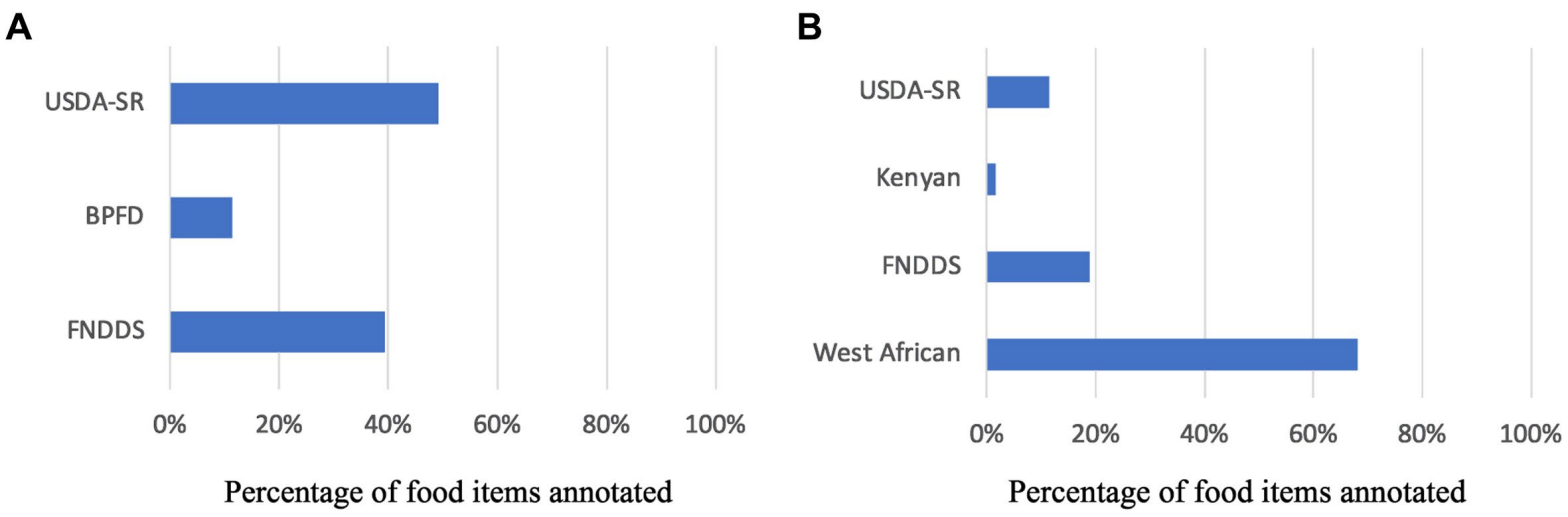
Food databases used in the **(A)** US and **(B)** Ghana studies.

A total of 231 eating episodes (99 on day 1 and 132 on day 2) were annotated in the US study. In Ghana, a total of 83 eating episodes were annotated. The number of food databases used in the US and Ghana studies was 3 and 4, respectively, for the annotation of 375 and 94 different food items, respectively. Furthermore, USDA-SR-annotated foods accounted for 50% of the food items annotated in the US study, followed by 40 and 10% from FNDDS and BPFD, respectively. In the Ghana study, however, West African food databases were frequently used (almost 70% of food items annotated), whereas USDA-SR, FNDDS, and Kenyan were used much less often (Figure 4). The average time to annotate each eating episode is 359±161 second ( 05:59±2:41inmm:ss ). Similarly, the average time to annotate a day is 1093±463 second ( 18:13±7:43inmm:ss ).

## 4. Discussion

The main objective of this paper was to provide a description of the semi-automated tool for annotating visual information to nutrient information, such as calories and key nutrients. The I2N tool is specifically designed to operate with a passive capture method, where images are captured without any user intervention. In contrast, traditional methods typically rely on an active capture method, where users are responsible for capturing the images themselves. I2N was created primarily for egocentric image capturing via wearable sensors, specifically for the AIM-2. I2N, however, can be extended to other image-capturing devices that may or may not have sensors. It could also be used for nutritional analysis of food records and 24-h dietary recalls that do not contain images. Eventually, I2N can be used with any model of automatic food intake detection as described in Ghosh and Sazonov (10) if the wearable sensor captures images. A limitation of I2N is that currently, I2N only runs on Windows 10 and above operating systems, but additional versions could potentially be developed for Mac OS and Linux.

One of the major advantages of I2N is that it reduces the number of images included in the full dataset that must be examined. While images may have been captured during non-eating and eating, the goal for nutritional analysis would be to examine only images that were taken while eating, thus saving time and effort from having to review all images collected. We implemented a sensor-guided image review to accomplish this goal. In the US study, we found that the AIM-2 captured 3009±354 (mean ± standard deviation) images every day, with 204 of those being food images on average (detected by the sensor using the machine learning model). As a result, this program minimizes the amount of human work and the number of images that needed to be reviewed. Furthermore, the link between the SDEEp plot and the thumbnail image simplified the annotation procedure. In the Ghana study, we found a similar statistic (a total of 1841±895 images each day, with an average of 267 food images). Thus, one of I2N’s main features is that sensor-guided image reviewing improves the efficiency of nutritional annotation of images.

Having a large and up-to-date food database is important for being able to analyze the wide-ranges of foods consumed by a population. A unique feature of I2N is that it allows for access to multiple food and nutrient databases and provides a wide choice of food items that are required in real-life situations. This feature is advantageous because research participants may eat food from a number of sources, including restaurants, branded items, and self-prepared meals. All of the food items from numerous sources are not contained in a single database. Branded food items, for example, are not included in the USDA-SR database. Furthermore, if the individual is not from the United States, the food database from that nation can be very useful in annotating energy consumption. The West African food database was used the most in the Ghana study, for example. To our knowledge, no other nutritional analysis software allows for simultaneous access to multiple food and nutrient databases. Additionally, I2N can be loaded with any food and nutrient database desired, provided the database is available in comma-separated values (CSV) or Microsoft Excel spreadsheet format.

I2N also determines (with the help of the annotator’s review) if a sensor-detected eating episode is true or false. I2N provides eating episode start and end times which can be manually entered, or automatically entered by linking the eating episode in the nutritional analysis panel with selected images the annotator determines at the beginning and end of each eating occasion. By doing so, the time stamp data will automatically populate in the appropriate fields. These details can be utilized to create a machine-learning model for detecting eating episodes using the annotated eating episode, AEEp information. On top of that by reviewing all the images, this software can be used to identify possible undetected eating episodes that were missed by the automatic food intake model. Furthermore, I2N can be used as a manual annotation tool for dietary intake, as well as a trained model to automatically estimate energy intake by utilizing consumed energy information.

The magnitude and sources of error in nutrient intake estimation using I2N are reported in Doulah et al. (8). The study calculated the error by comparing with the researcher-conducted weighed food record. It found two main sources of errors: 1) food identification errors and 2) portion size errors, with errors of 4.7 and 32.5% for food identification and portion size, respectively. Common to all image-based methods, food identification errors may stem from misclassification of the food items or inability to differentiate between visually similar foods and beverages that appear the same. For instance, an annotator may have difficulty distinguishing between regular, diet, and alcohol-added cola. To aid in food identification, an annotator could use external information such as from a questionnaire that asks study participants about usual food intake habits such as type of soda and type of milk. Portion size estimation errors are inherent to the 2D imaging, as detailed in the review (24). Another limitation of I2N is that while I2N tool can handle data without any restrictions on the number of days or participants and significantly reduce manual labor, it cannot completely eliminate the need for nutritionist’s time. In the future, we plan to automate food identification and portion size estimation by utilizing methods of computer vision.

## 5. Conclusion

We developed a semi-automated image-to-nutrient (I2N) software tool for nutritional analysis of food and beverages seen in images. An annotator uses visual information to enter food items and portion sizes to calculate energy and nutrient intake. I2N provides sensor-guided image review, which in the studies evaluated here, reduced the total number of images by at least 85%. I2N also marked a sensor detected eating episode (SDEEp) as true or false. For annotation, I2N used multiple food databases, including both American and African food databases. Multiple food databases have been shown to be useful in annotating food items in real-life scenarios. Furthermore, using both the tool and the wearable sensor system for energy intake estimation eliminates the need for self-reporting and memorization. I2N is a unique tool that allows for simultaneous viewing of food images, sensor-guided image review, and access to multiple databases in one tool, making nutritional analysis of food images efficient. I2N has the flexibility to be used with images from different devices, or to be used for nutritional analysis of dietary data without images, such as estimated or weighed food records, and 24 h dietary recalls. In the future, food identification and portion size estimation can be automated through the utilization of computer vision algorithms.

## Data availability statement

The original contributions presented in the study are included in the article/Supplementary material, further inquiries can be directed to the corresponding author.

## Ethics statement

The studies involving human participants were reviewed and approved by The University of Alabama’s Institutional Review Board, Human Subject Institutional Review Boards of the University of Georgia and the University of Ghana. The patients/participants provided their written informed consent to participate in this study.

## Author contributions

TG and ES developed the tool and wrote the paper. TM, JH, and CD used the tool to annotate study data and revise the paper. MM used the tool for annotation and revised the paper. AA, WJ, BL, GF, MS-A, TB, and MS conducted the Ghana study to collect data. All authors contributed to the article and approved the submitted version.

## Funding

This work was supported by the National Institutes of Health under Award number R01DK100796, UL1TR002535 and the Bill and Melinda Gates Foundation (Contract ID: OPP1171395). The content is solely the responsibility of the authors and does not necessarily represent the official views of the National Institutes of Health.

## Conflict of interest

MM serves on the Avocado Nutrition Science Advisory for the Hass Avocado Board.

The remaining authors declare that the research was conducted in the absence of any commercial or financial relationships that could be construed as a potential conflict of interest.

## Publisher’s note

All claims expressed in this article are solely those of the authors and do not necessarily represent those of their affiliated organizations, or those of the publisher, the editors and the reviewers. Any product that may be evaluated in this article, or claim that may be made by its manufacturer, is not guaranteed or endorsed by the publisher.
