# Supplementary material for: I2N: image to nutrients, a sensor guided semi-automated tool for annotation of images for nutrition analysis of eating episodes
Source: Front Nutr. 2023 Jul 27;10:1191962. doi: 10.3389/fnut.2023.1191962 (PMC10415029; doi:10.3389/fnut.2023.1191962)
Supplement: Supplementary file 1 [file Data_Sheet_1.docx]

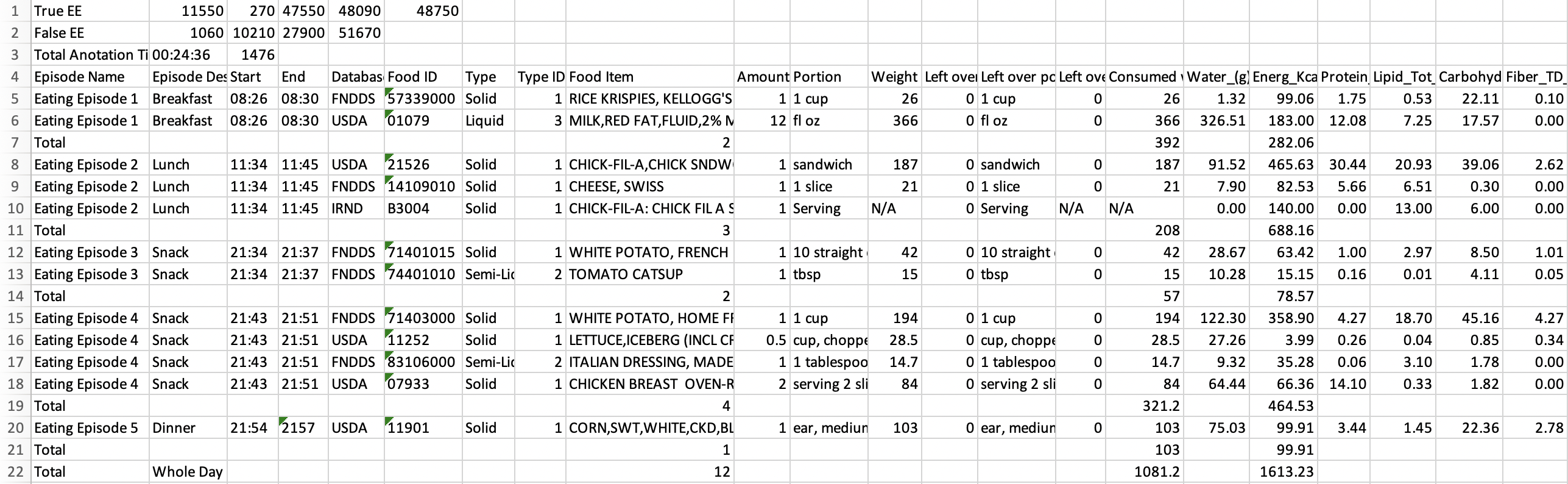


(a)


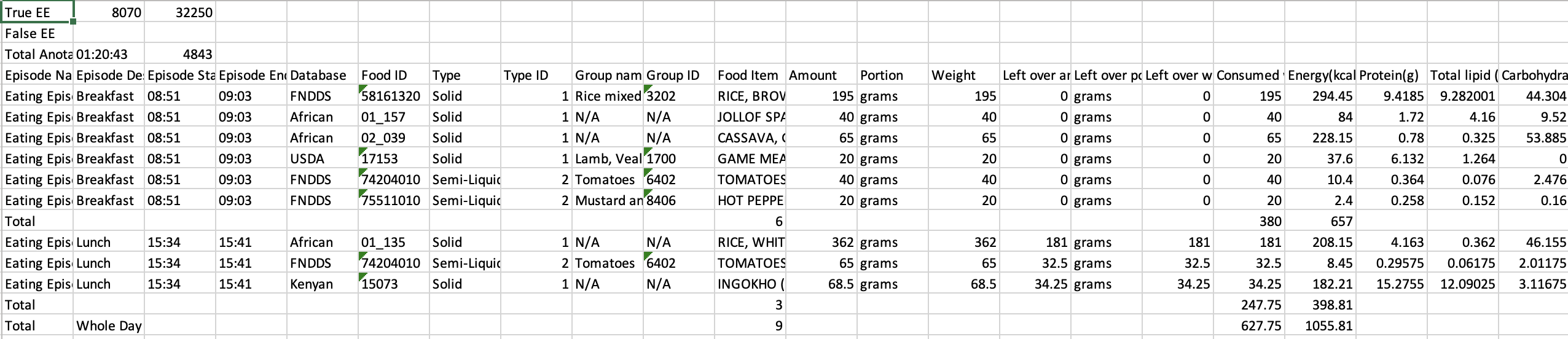


(b)

Figure: Example annotation output with eating episodes and nutrients from (a) US study, (b) Ghana study.
